# Integrated Extraction Optimization and HPLC‐based Quality Evaluation of Thiophenes from *Tagetes erecta* Roots

**DOI:** 10.1002/ansa.70055

**Published:** 2025-11-17

**Authors:** Shuo Tian, Sainan Li, Jisu Park, Jong‐Sup Bae, MinKyun Na

**Affiliations:** ^1^ College of Pharmacy Chungnam National University Daejeon Republic of Korea; ^2^ College of Pharmacy Kyungpook National University Daegu Republic of Korea

**Keywords:** chromatographic fingerprint, extraction optimization, HPLC quantification, *Tagetes erecta*, thiophenes

## Abstract

*Tagetes erecta* L. is widely studied for its flower‐derived lutein, which is known for promoting eye health. However, its roots contain uniquely thiophenes—absent from the flowers and leaves—which exhibit valuable bioactivity. Our recent study has reported on patent applications related to their efficacy in modulating benign prostatic hyperplasia, underscoring their pharmaceutical and functional potential. Despite this, no optimized extraction or quality evaluation of thiophene‐rich *T. erecta* roots has been reported. This study aimed to establish a reliable extraction strategy for thiophene‐rich *T. erecta* roots and to ensure chemical consistency through validated HPLC quantification and chromatographic fingerprint similarity analysis. Three major thiophenes—5‐(4‐hydroxybut‐1‐ynyl)‐2,2'‐bithiophene (**1**), 5‐(4‐acetoxybut‐1‐ynyl)‐2,2'‐bithiophene (**2**) and 5‐(3‐buten‐1‐ynyl)‐2,2'‐bithiophene (**3**)—were isolated and structurally confirmed. The extraction variables (solvent, plant part, method, sample amount and time) were optimized. Quantification was performed via validated HPLC‐PDA using in‐house purified standards (≥ 98%), and fingerprint similarity was evaluated. Ultrasonic extraction for 2 h with 5 g of root powder in 95% ethanol gave optimal yield and reproducibility. The HPLC method exhibited excellent linearity (*R* ^2 ^>  0.999), precision (RSD < 1%) and recovery (93.20%–105.24%). Fingerprint analysis of 13 common peaks revealed high similarity (0.984–1.000), indicating stable chemical profiles. This study provides a validated workflow for thiophene‐rich *T. erecta* roots, highlights the significance of root‐derived thiophenes and offers a practical basis for process development, quality control and future standardization of thiophenes and related constituents.

## 1 Introduction

*Tagetes erecta* L., a member of the Asteraceae family, is widely cultivated in tropical and subtropical regions and is valued both as an ornamental plant and as a source of bioactive metabolites with medicinal and industrial applications [1]. Traditionally, it has been employed for its antibacterial, antioxidant and anti‐inflammatory properties [2, 3], which derive from its diverse secondary metabolites, including thiophenes, flavonoids, carotenoids and triterpenoids. Thiophenes, a distinct class of sulphur‐containing polyacetylenes, possess broad‐spectrum biocidal activities, such as antifungal, nematocidal and insecticidal effects, and are predominantly concentrated in the roots of *T. erecta* [4, 5, 6]. Our recent study demonstrated that *T. erecta* root‐derived thiophenes modulate benign prostatic hyperplasia (BPH), resulting in patent applications [7, 8].

Recently, sequential optimization strategies have gained increasing attention in natural product research, offering greater reproducibility and efficiency compared with traditional empirical approaches. This is particularly important for complex herbal matrices, where even slight variations in parameters such as solvent type, extraction method and sample amount can markedly influence chemical outcomes [9, 10]. Ethanol is generally preferred for thiophene extraction because its polarity is suitable for nonpolar to moderately polar compounds; however, prolonged heating can cause degradation, necessitating careful optimization [11, 12].

Although thiophenes from *T. erecta* have been extensively studied for their bioactivity and chemical structures [13, 14], few studies have addressed their quantification under optimized extraction conditions [15, 16]. Existing methods often fail to effectively integrate extraction optimization with chemical analysis, which limits their reliability for quality evaluation.

To overcome these limitations, the present study employed high‐performance liquid chromatography (HPLC), a widely accessible analytical platform [17, 18, 19], to establish an integrated workflow that combines extraction optimization with validated quantification using in‐house purified thiophene standards and fingerprint similarity analysis. This framework provides analytical baselines for *T. erecta* root‐derived thiophenes, thereby ensuring reproducible characterization and supporting their development as standardized phytochemical resources.

## 2 Experimental

### 2.1 Plant Material

The dried roots, leaves and flowers of *T. erecta* were purchased from Woori Green Science Company (Korea) in 2022 and identified by Prof. MinKyun Na (College of Pharmacy, Chungnam National University, Daejeon, Korea). Voucher specimens (CNU‐TR‐202303, CNU‐TL‐202208 and CNU‐TF‐202303) were deposited in the Pharmacognosy Laboratory of the College of Pharmacy, Chungnam National University. The plant materials were air‐dried in shade before storage, unless stated otherwise.

*T. erecta* roots were collected from plants cultivated under controlled hydroponic conditions, and a single batch was used to ensure material consistency throughout the optimization experiments.

### 2.2 Instrumentation

Nuclear magnetic resonance (NMR) experiments were conducted using a Bruker AVANCE Neo 400 spectrometer (^1^H, 400 MHz; ^13^C, 100 MHz). Liquid chromatography–mass spectrometry (LC–MS) data were obtained using a SYNAPT G2 mass spectrometer (Waters, Manchester, UK). Thin‐layer chromatography (TLC) was performed on glass plates precoated with silica gel 60 F_254_ and RP‐18 F_254_ (20 cm × 20 cm, 200 µm, 60 Å; Merck, Kenilworth, NJ, USA). Medium‐pressure liquid chromatography (MPLC) was conducted using a Biotage Isolera apparatus equipped with a reversed‐phase C_18_ column (Biotage, Uppsala, Sweden). Preparative HPLC was carried out on a Gilson system (PLC 2020) at a flow rate of 7.0 mL/min using a Hector M C_18_ column (250 mm × 21.2 mm, 5 µm). Analytical HPLC was performed on a Hector M C_18_ column (250 mm × 4.6 mm, 5 µm) equipped with a Shimadzu SPD‐M20A photodiode array (PDA) detector.

### 2.3 Isolation and Identification of Standards

Dried roots of *T. erecta* (1.0 kg) were extracted with 95% ethanol (36 L) at room temperature for 7 days. The combined extracts were concentrated under reduced pressure to yield a crude extract (39.23 g), which was subjected to MPLC (Biotage SNAP Cartridge KP‐C_18_‐HS, 400 g) using a MeCN–H_2_O gradient (30%–100%) with UV detection at 340 nm (flow rate: 100 mL/min), yielding four fractions (Fr.1–Fr.4). Fr.3 (3.03 g) was further separated by MPLC (Biotage SNAP Cartridge KP‐C_18_‐HS, 120 g) with a 60%–100% MeCN–H_2_O gradient to yield seven subfractions (Fr.3.1–Fr.3.7). Compound **1** (24.1 mg, *t*_R_ = 33.0 min) was isolated from Fr.3.3 (246.4 mg) by preparative HPLC (Hector C_18_, 250 mm × 21.2 mm, 5 µm, 7 mL/min) using isocratic elution with 75% MeOH. Compound **2** (363.5 mg, *t*_R_ = 34.0 min) was purified from Fr.3.4 (420.1 mg) under the same chromatographic conditions. Compound **3** (58.0 mg, *t*_R_ = 41.0 min) was obtained from Fr.3.6 (204.6 mg) by HPLC (Hector C_18_, 250 mm × 21.2 mm, 5 µm, 7 mL/min) using 85% MeOH.

**FIGURE 1:**
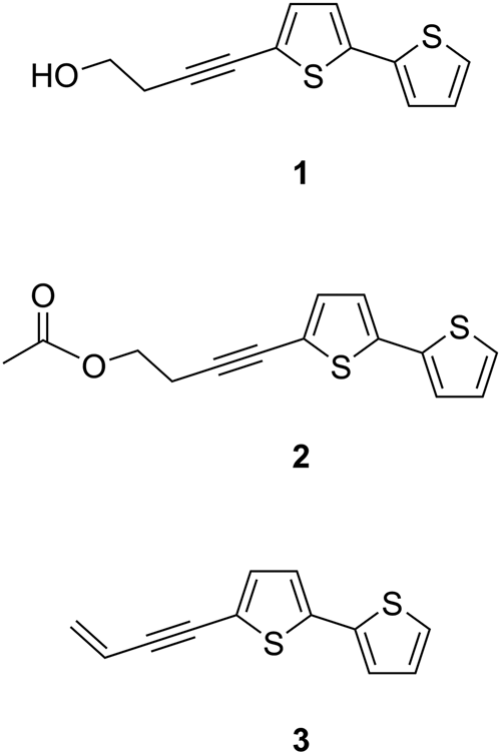
Chemical structures of compounds **1**–**3** isolated from *T. erecta* roots.

The structures of the three compounds, 5‐(4‐hydroxybut‐1‐ynyl)‐2,2'‐bithiophene (**1**), 5‐(4‐acetoxybut‐1‐ynyl)‐2,2'‐bithiophene (**2**) and 5‐(3‐buten‐1‐ynyl)‐2,2'‐bithiophene (**3**) were identified by LC–MS, ^1^H‐ and ^13^C‐NMR spectroscopy data and confirmed by comparison with previous reports [20, 21, 22] (Figure 1). The NMR spectra are provided in the Supporting Information. All three compounds were confirmed to be ≥ 98% pure by HPLC analysis and were subsequently used as reference standards for quantitative analysis.

Compound **1**: yellow oil; ^1^H and ^13^C NMR data: see Table S1; ESI‐MS *m/z* 235.0254 [M + H]^+^.

Compound **2**: yellow oil; ^1^H and ^13^C NMR data: see Table S1; ESI‐MS *m/z* 277.0357 [M + H]^+^.

Compound **3**: yellow oil; ^1^H and ^13^C NMR data: see Table S1; ESI‐MS *m/z* 217.0142 [M + H]^+^.

### 2.4 Preparation of Standards

Stock solutions of compounds **1**–**3** were prepared in methanol at 1 mg/mL. Calibration standards were individually prepared at various concentrations corresponding to their detection ranges (**1**: 1.0–8.0, **2**: 15.0–120 and **3**: 5.0–80 µg/mL). All standard solutions were freshly prepared, filtered through 0.20 µm syringe filters (Dismic‐13JP, Advantec, Japan) before injection and then analysed within 24 h.

### 2.5 Sample Preparation

Dried *T. erecta* root, flower and leaf powders were accurately weighed (1–10 g, depending on the experiment) and extracted with 50 mL of 95% ethanol using three different methods: ultrasonic extraction, reflux extraction and room‐temperature maceration. After extraction, the mixtures were cooled to room temperature, transferred to 50 mL volumetric flasks and adjusted to volume with 95% ethanol. The extracts were filtered through 0.20 µm syringe filters (Dismic‐13JP, Advantec, Japan) and directly subjected to HPLC without an internal standard.

### 2.6 Extraction Parameter Optimization

Based on the standardized sample preparation method (Section 2.5), a sequential optimization strategy was adopted to establish a reliable protocol for extracting thiophenes from *T. erecta* roots. This practical approach, rather than relying on complex statistical modelling, is directly applicable to phytochemical quality evaluation.

The optimization consisted of two stages: preliminary screening of key extraction variables and stepwise optimization of the critical parameters. The screened variables included the plant part, solvent type and extraction method, while the optimization parameters were the sample amount and extraction time. The extraction efficiency was evaluated from the average HPLC peak areas of the three major thiophenes **1**–**3** to maximize the yield and ensure reproducibility. For comparison among compounds with different detector responses, the peak areas were normalized by dividing by the maximum value for each compound across the dataset prior to plotting. Standard errors of the mean (SEM) were calculated from replicate values after normalization.

#### 2.6.1 Preliminary Screening of Extraction Variables

To establish baseline conditions for subsequent optimization, preliminary screening experiments were performed for three variables: plant part, solvent type and extraction method. Each test used 1.0 g of powdered sample. First, the distribution of thiophenes in different plant parts (roots, leaves and flowers) was compared using 95% ethanol, which was identified previously as an effective solvent. Reflux extraction was employed here as a standardized baseline for comparative analyses. Next, distilled water and 95% ethanol were used in reflux extraction for 3 h to confirm the efficiency of ethanol. Finally, three extraction methods were compared under different time conditions: reflux (1, 2 and 3 h), ultrasonic extraction (30 min, 1, 2 and 3 h) and maceration (3 days). The temperature conditions were method‐dependent: reflux at ∼78°C (the boiling point of 95% ethanol), ultrasonication at 22°C–53°C (owing to heat transfer from the water bath) and maceration at ambient temperature (without external heating). These temperatures were controlled within each method to ensure compound stability and comparability. Extraction efficiency in each test was evaluated based on the HPLC peak areas of compounds **1**–**3**.

#### 2.6.2 Stepwise Optimization of Sample Amount and Extraction Time

Based on the screening results, ultrasonic extraction with 95% ethanol for 2 h was selected as the reference condition for further optimization. The sample amount (1–10 g) and extraction time (30–180 min) were varied while keeping other conditions (e.g., solvent type) constant. Repeatability was evaluated by performing extractions on three consecutive days, with three replicate HPLC injections each day. Short‐term stability was assessed by reanalysing extract solutions stored at 4°C for 24 and 48 h.

### 2.7 HPLC Analysis

HPLC analysis of thiophenes was carried out on a Shimadzu system equipped with a PDA detector (SPD‐M20A) and Hector M C_18_ column (250 mm × 4.6 mm, 5 µm). The mobile phase of water (A) and acetonitrile (B) was delivered at a flow rate of 0.8 mL/min under a linear gradient from 30% to 100% B over 60 min. The column temperature was 30°C, and UV detection was performed at 340 nm.

Compounds **1**–**3** were identified by comparing their retention times and UV spectra with those of in‐house purified standards, which were structurally confirmed by NMR and LC–MS. Calibration curves were constructed from five concentrations of each compound analysed in triplicate. Each calibration sample was prepared from an independent standard solution to ensure accuracy and reproducibility. Linearity was evaluated by plotting concentration against peak area, with all compounds showing excellent correlation with *R* ^2^ values above 0.999. The limits of detection (LOD) and quantification (LOQ) were calculated according to IUPAC guidelines based on the standard deviation of the response and the slope of the calibration curve, respectively [23].

### 2.8 Statistical Analysis

Differences among extraction conditions were analysed using one‐way ANOVA followed by Tukey's multiple comparison test (*p* < 0.05) in OriginPro 2023. Data are expressed as the mean ± SEM (*n* = 3 or 9).

### 2.9 Method Validation

System suitability was evaluated using six replicate injections of the standard solution of compound **2**, which was selected as the representative analyte owing to its strong and stable chromatographic response. Method validation followed IUPAC and general analytical guidelines to ensure the reliability of thiophene quantification. The validation parameters included linearity, sensitivity, precision and accuracy. Linearity for compounds **1**–**3** was evaluated over the ranges of 1.0–8.0, 15.0–120.0 and 5.0–80.0 µg/mL, respectively, with *R* ^2^ values calculated using OriginPro 2023. Sensitivity was expressed based on the LOD and LOQ, calculated as LOD = 3*σ*/*S* and LOQ = 10*σ*/*S*, respectively, where *σ* is the standard deviation of the response and *S* is the slope of the calibration curve. Precision was determined from repeatability (intra‐ and inter‐day variability) based on triplicate analyses. Accuracy was determined by spiking the pre‐extracted root matrix at three concentration levels (80%, 100% and 120%) and calculating the recovery.

### 2.10 Solution Stability

Standard solutions of compounds **1**–**3** were reanalysed after 24 and 48 h of storage at 4°C and compared with freshly prepared solutions to assess short‐term stability.

### 2.11 Chromatographic Fingerprint and Similarity

#### 2.11.1 Identification of Characteristic Peaks

HPLC chromatograms of *T. erecta* root samples obtained under each extraction condition were acquired using the parameters described in Section 2.7. All samples were prepared from the same batch of root powder to ensure comparability.

Chromatographic data were processed using the Similarity Evaluation System for Chromatographic Fingerprints of Traditional Chinese Medicine (Version 2004A; Chinese Pharmacopoeia Committee). The chromatogram of the extract obtained by ultrasonication for 2 h offered the best quantitative performance and was therefore selected as the reference. All chromatograms were aligned to this reference by multipoint retention‐time correction using the peaks of compounds **1**–**3** as anchors. Thirteen common characteristic peaks were identified across all chromatograms and used for similarity analysis.

#### 2.11.2 Similarity Analysis

Similarity among chromatograms was evaluated based on the relative peak areas and Pearson correlation coefficients. The relative peak areas were calculated as percentages of the total peak area:

$$\%\mathrm{Peak}\;\mathrm{area}\;=\frac{\mathrm{peak}\;\mathrm{area}\;\mathrm{of}\;\mathrm{each}\;\mathrm{peak}}{\mathrm{total}\;\mathrm{peak}\;\mathrm{area}\;\mathrm{of}\;\mathrm{all}\;\mathrm{peaks}}\;\times \;100$$

The similarity between each chromatogram and the reference was expressed as Pearson's correlation coefficient (*r*):

$$r=\frac{\sum_{i=1}^{n}\left(x_{i}-\bar{x}\right)\left(y_{i}-\bar{y}\right)}{\sqrt{\sum_{i=1}^{n}{\left(x_{i}-\bar{x}\right)}^{2}\sum_{i=1}^{n}{\left(y_{i}-\bar{y}\right)}^{2}}}$$

where $x_{i}$ and $y_{i}$ represent the areas of the *i*‐th characteristic peak in the test and reference chromatograms, respectively, and $\bar{x}$ and $\bar{y}\;$ are their means. The resulting similarity indices were used to assess the chemical consistency across extracts.

#### 2.11.3 Chemical Pattern Recognition of HPLC Fingerprints

Visual comparison of aligned chromatograms revealed no substantial differences in peak shape, distribution or resolution; most samples exhibited highly similar profiles. Minor variations in peak intensity were observed but were insufficient for reliable quality discrimination. Therefore, visual inspection served only as a supplementary step, whereas the similarity indices described in Section 2.11.2 provided a more objective and reproducible assessment of chemical consistency.

## 3 Results and Discussion

### 3.1 Extraction Parameter Optimization

#### 3.1.1 Plant Part, Solvent Type and Extraction Method

To establish a rational extraction protocol for thiophenes from *T. erecta* roots, three variables were initially screened: plant part, solvent type and extraction method. Compounds **1**–**3** were detected exclusively in root extracts, with no corresponding peaks in leaf or flower extracts prepared under identical conditions (Figure S1). This confirmed that thiophenes accumulated specifically in the roots of *T. erecta*, which is consistent with previous reports [24, 25].

To evaluate the solvent performance, extracts were prepared using ethanol and water under identical reflux conditions for 3 h. Thiophenes were detected only in the ethanol extracts (Figure S2), suggesting a polarity mismatch between water and the thiophenes, which are moderately lipophilic sulphur‐containing polyacetylenes. These results supported the selection of ethanol as the extraction solvent [26].

**FIGURE 2:**
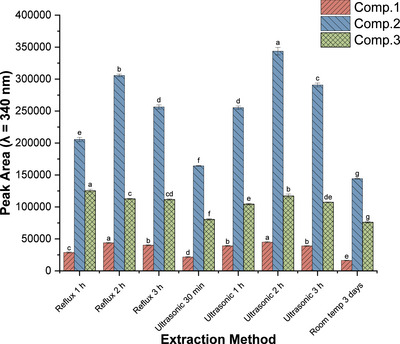
Extraction efficiency of compounds **1**–**3** using different methods (reflux, ultrasonication and room‐temperature maceration). Data are mean ± SEM (*n* = 3). Different letters for the same compound indicate significant differences between means (*p* < 0.05).

Finally, three extraction methods (reflux, ultrasonication and room‐temperature maceration) at different extraction times were compared using 1 g of root powder (Figure 2). Ultrasonic extraction for 2 h yielded the highest overall peak intensities for all three compounds, whereas room‐temperature maceration was markedly less effective. Reflux yielded moderate responses. Thus, ultrasonic extraction offers an optimal balance between efficiency and practicality and was selected for further optimization.

#### 3.1.2 Sample Amount

**FIGURE 3:**
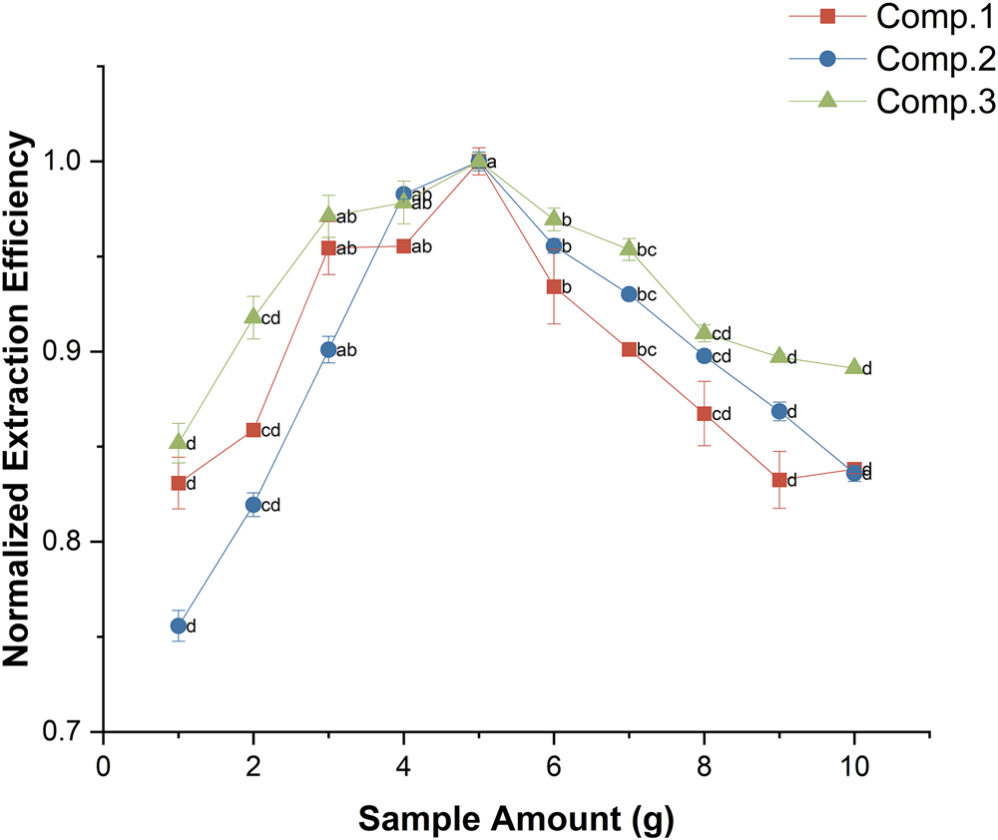
Normalized extraction efficiency of compounds **1**–**3** using different sample amounts. Data are mean ± SEM (*n* = 3), calculated from normalized replicate values. Different letters for the same compound indicate significant differences between means (*p* < 0.05).

To investigate the effect of sample amount, *T. erecta* root powders at 10 levels (1–10 g, 1 g increments) were extracted by ultrasonication with 95% ethanol for 2 h. As shown in Figure 3, the extraction efficiency increased progressively from 1 to 5 g. Beyond 5 g, the efficiency declined, despite larger samples showing higher absolute peak areas. This reduction in efficiency was likely due to limited solvent accessibility and mass‐transfer constraints at higher solid loadings. Similar trends have been reported in other plant extraction studies, where dense matrices hindered solvent penetration and reduced extraction efficiency [16, 27]. Overall, the 5 g sample provided the best balance between extraction efficiency, signal intensity and reproducibility, and was therefore selected for subsequent experiments.

#### 3.1.3 Extraction Time

**FIGURE 4:**
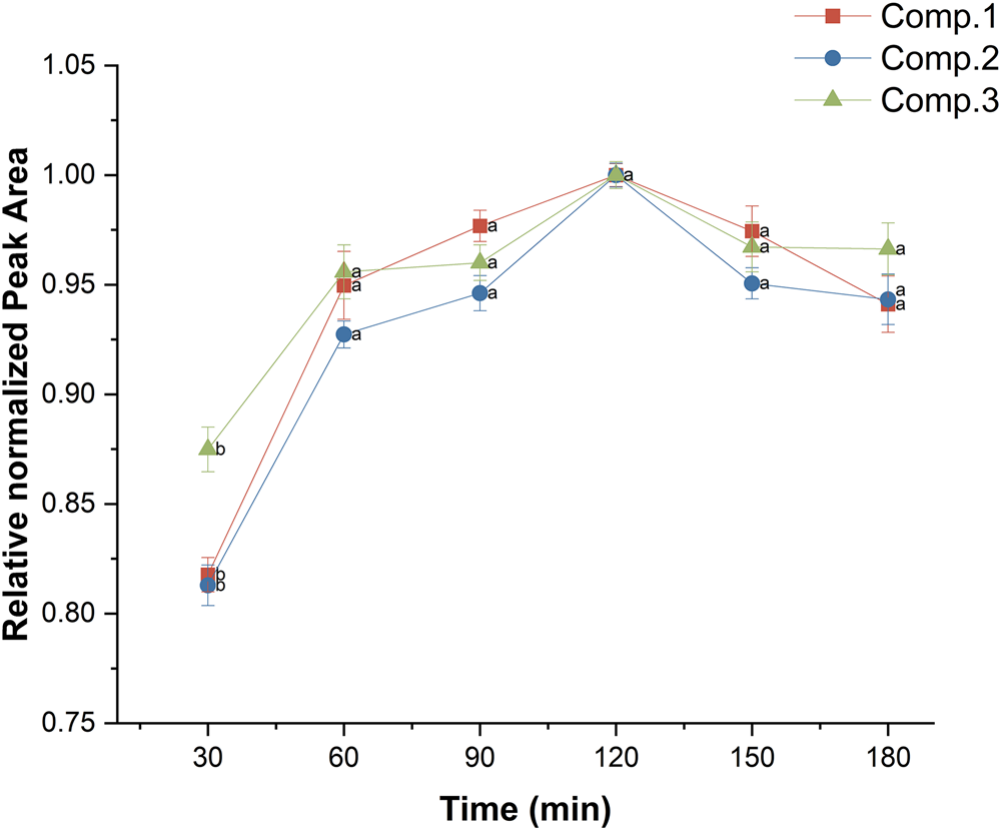
Normalized extraction efficiency of compounds **1**–**3** using different extraction times. Data are mean ± SEM (*n* = 9), calculated from normalized replicate values. Different letters for the same compound indicate significant differences between means (*p* < 0.05).

To evaluate the effect of extraction time, ultrasonication was performed at six time points (30, 60, 90, 120, 150 and 180 min) using 5 g of root powder under identical conditions. As shown in Figure 4, the peak areas of compounds **1**–**3** increased steadily from 30 to 120 min, indicating that the extraction efficiency improved over time. A significant improvement was observed between 30 and 120 min (*p* < 0.05), particularly for compound **2**, whose peak area increased by approximately 20.6%. By contrast, no significant differences (*p* > 0.05) were observed between 120 and 180 min, indicating that equilibrium was reached by 120 min. The peak areas plateaued or declined slightly after 120 min, suggesting no further benefit from prolonged extraction. For example, compound **1** showed a 5.2% decrease at 180 min compared with its maximum at 120 min, indicating potential degradation or saturation. Therefore, the optimal extraction time was 120 min, which provided the highest yield and statistical stability. These results were consistent across three independent experiments conducted on different days. For extracts prepared by ultrasonication for 120 min, the relative standard deviations (RSDs) were below 3% for all three compounds, indicating good repeatability. In addition, no significant degradation or retention‐time shift was observed after storage at 4°C for 24 or 48 h, confirming short‐term chemical stability (Table 1). Overall, ultrasonic extraction for 120 min is effective for achieving a high yield, good reproducibility and short‐term stability of thiophenes.

**TABLE 1:** Precision of compounds **1**–**3** under the optimized ultrasonic extraction condition (5 g, 2 h).

Compound	Inter‐day RSD (%)	Intra‐day RSD (%)		
		Day 1	Day 2	Day 3
**1**	1.49	0.56	0.35	0.54
**2**	1.32	2.32	0.34	0.07
**3**	2.14	0.49	1.84	2.28

### 3.2 HPLC Method Validation

System suitability testing confirmed that the developed HPLC method met all performance criteria. The representative peak of compound **2** had a theoretical plate number of 3744 (> 2000) and tailing factor of 1.2 (< 2.0), indicating satisfactory column efficiency and peak symmetry. These results verified the suitability of the system for reliable separation and quantification of thiophene derivatives.

**FIGURE 5:**
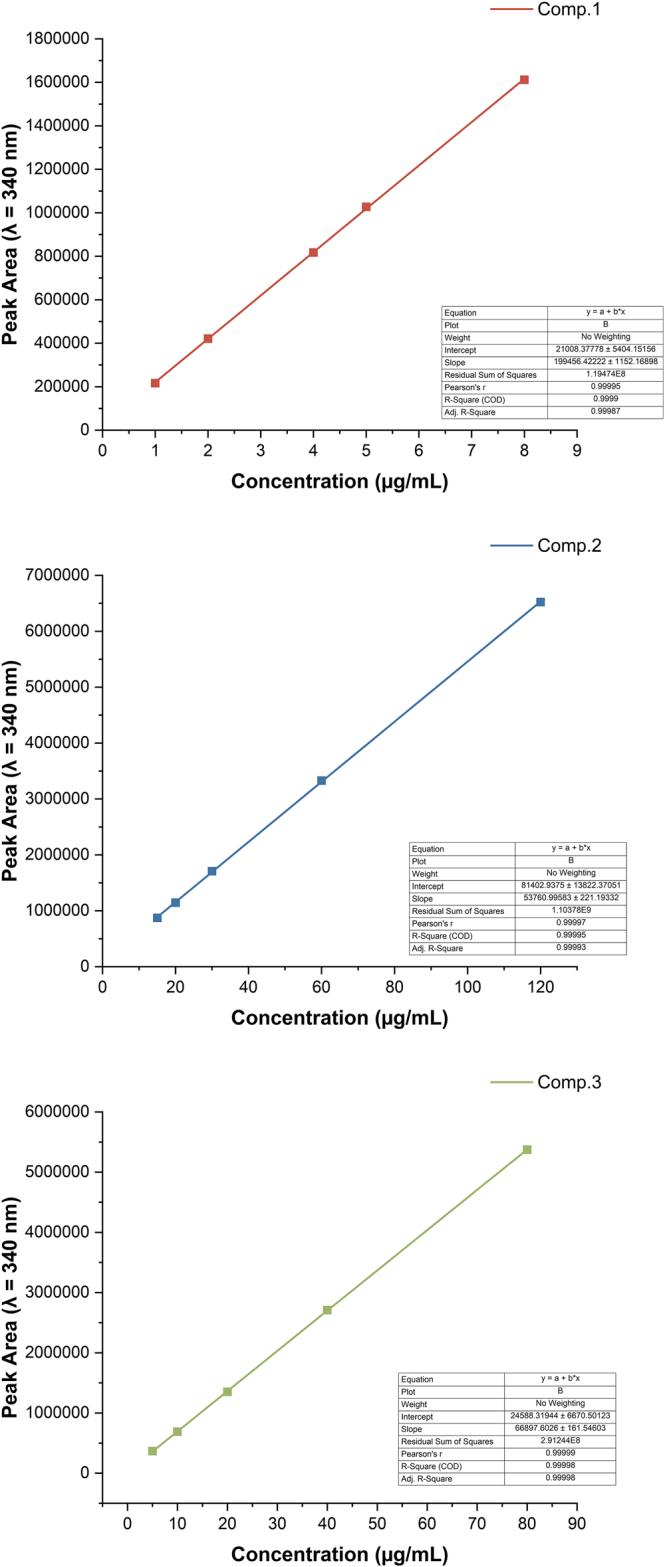
Standard calibration curves of compounds **1**–**3** based on HPLC peak areas at 340 nm. Data are mean ± SD (*n* = 3).

The method demonstrated reliable performance across all validation parameters. All three compounds showed strong linearity, with correlation coefficients (*R* ^2^) consistently above 0.999, as illustrated by the calibration curves in Figure 5. The method exhibited high sensitivity, and precision was confirmed by low RSD values across concentrations and test days, demonstrating excellent repeatability and reproducibility. Recovery experiments using standard addition yielded values between 93.2% and 105.2%, confirming the accuracy and robustness of the method for quantification. Detailed validation results are given in Table 2.

**TABLE 2:** Validation parameters of the HPLC method for compounds **1**–**3**.

Parameter	Compound 1	Compound 2	Compound 3
Linearity range (µg/mL)	1.0–8.0	15.0–120.0	5.0–80.0
Regression equation	*y* = 199456.42*x *+ 21008.38	*y *= 53760.99*x *+ 81402.94	*y *= 66897.60*x *+ 24588.32
*R* ^2^	0.99995	0.99993	0.99998
LOD (µg/mL)	0.10	1.18	0.49
LOQ (µg/mL)	0.32	3.57	1.47
Precision (RSD, %)	Intra‐day: 0.17–0.70 Inter‐day: 0.18–0.85	Intra‐day: 0.12–0.61 Inter‐day: 0.45–0.92	Intra‐day: 0.24–0.69 Inter‐day: 0.14–0.83
Accuracy (Recovery, %)	93.20–103.37	95.33–101.78	98.71–105.24

### 3.3 Solution Stability

Standard solutions of compounds **1**–**3** stored at 4°C for 24 and 48 h retained 96.48%–100.73% and 97.04%–100.62% of their initial peak areas, respectively, with all RSDs below 2%. No significant changes in retention time or peak shape were observed, indicating that the standards remained chemically stable for at least 48 h under refrigerated conditions and could be reliably used for quantitative analysis (Table S2).

### 3.4 Chromatographic Fingerprint and Similarity Evaluation

Fingerprint chromatograms were acquired using a 30%–100% acetonitrile gradient completed within 40 min, which had been established through preliminary optimization as providing an optimal balance between separation efficiency and run time.

#### 3.4.1 Identification of Characteristic Peaks

Chromatograms of extracts obtained under different conditions were subjected to fingerprint analysis, with the extract produced by ultrasonication for 2 h selected as the reference. Thirteen characteristic peaks common to all chromatograms were identified for similarity evaluation. Based on their UV absorption spectra (*λ*_max_ ≈ 340 nm), these peaks were attributed to thiophene‐type polyacetylenes, which constitute the major secondary metabolites in *T. erecta* root extracts.

#### 3.4.2 Similarity Analysis

Similarity indices were calculated from the relative peak areas of the 13 characteristic peaks. The similarity values ranged from 0.984 to 1.000 (Table S3), indicating a high degree of chemical consistency among the extracts. Although the indices were close to one, slight variations in peak intensity were observed, reflecting minor differences in relative abundance depending on the extraction conditions. This suggests that the method provides overall consistency and adequate sensitivity for capturing subtle compositional variations.

#### 3.4.3 Chemical Pattern Recognition of HPLC Fingerprints

**FIGURE 6:**
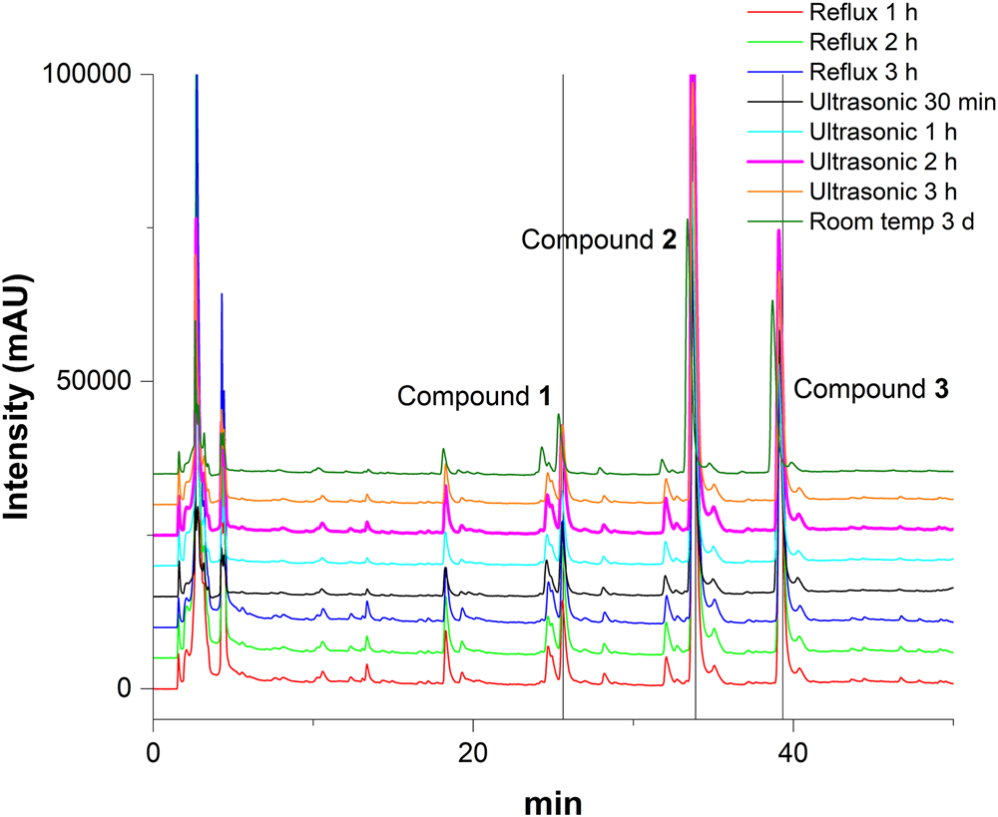
HPLC fingerprints of *T. erecta* root extracts obtained under different extraction conditions.

The overlay of HPLC chromatograms in Figure 6 shows that the peak patterns were consistent across extracts. Minor variations in peak intensity were observed, but high similarity indices (> 0.98) confirmed overall chemical consistency. Future studies should verify the chemical identities of these peaks using LC–MS.

The optimized extraction increased both thiophene yield and chemical consistency, which are critical for analytical reproducibility and for strengthening the reliability of subsequent biological evaluations. *T. erecta* root‐derived thiophenes exhibit antifungal, nematocidal and insecticidal effects and have been reported to modulate BPH [7, 8]. Therefore, improved extraction provides a practical foundation for pharmacological investigations aimed at validating their functional properties.

This work established a reliable workflow for thiophene extraction and quality evaluation from *T. erecta* roots. The thiophene composition may vary with plant origin or harvest season, as observed for other *Tagetes* metabolites. For example, the carotenoid and flavonoid levels in *Tagetes patula* flowers differ markedly depending on postharvest treatment, with ensilage yielding the highest contents (5.0–7.8 g kg^−1^ dw of carotenoids and 19–50 g kg^−1^ dw of flavonoids) [28]. The present optimization was performed using a single‐source batch cultivated under controlled conditions. Future studies should therefore include multi‐source and seasonal materials to confirm the robustness and broader applicability of the method.

Stepwise single‐factor optimization was adopted in this study because of its practicality, reproducibility and clear interpretability of individual factors such as solvent type, sample amount and extraction time. Its methodological transparency and operational simplicity provide a reliable basis for establishing reproducible extraction protocols. However, this approach has limited capacity to reveal potential interactions among variables. Multivariate optimization could further enhance the robustness of the extraction strategy.

## 4 Conclusion

This study established a practical and integrated framework for the standardized quality evaluation of *T. erecta* root‐derived thiophenes. Stepwise extraction optimization validated HPLC‐PDA quantification using in‐house purified standards (≥ 98%), and chromatographic fingerprint similarity analysis was combined to establish analytical baselines. The optimized extraction conditions (ultrasonication of 5 g of root powder with 95% ethanol for 2 h) provided high yield and repeatability. The HPLC method satisfied analytical requirements (*R* ^2^ > 0.999, RSD < 1%, recoveries 93.2%–105.2%, with appropriate LOD/LOQ), and fingerprint similarity across 13 characteristic peaks remained high (0.984–1.000), confirming stable profiling under varied extraction conditions.

Taken together, these results highlight the potential of thiophenes as root‐specific marker compounds of *T. erecta* and establish a robust platform for process development and quality control. This study provides a foundation for the standardization and industrial application of *T. erecta* roots and their constituent thiophenes, highlighting their potential as standardized resources for pharmaceutical and functional applications.

## Author Contributions

**Shuo Tian**: writing – original draft, methodology, investigation, formal analysis. **Sainan Li**: investigation. **Jisu Park**: investigation. **Jong‐Sup Bae**: investigation. **MinKyun Na**: writing – review and editing, conceptualization.

## Funding

This work was supported by the research fund of the Chungnam National University.

## Ethics Statement

The authors have nothing to report.

## Conflicts of Interest

The authors declare no conflicts of interest.

## Supporting information




**Supporting File**: ansa70055‐sup‐0001‐SuppMat.docx.

## Data Availability

The data that support the findings of this study are available from the corresponding author upon reasonable request.
