# Supplementary material for: Integrated Extraction Optimization and HPLC‐based Quality Evaluation of Thiophenes from Tagetes erecta Roots
Source: Anal Sci Adv. 2025 Nov 17;6(2):e70055. doi: 10.1002/ansa.70055 (PMC12623060; doi:10.1002/ansa.70055)
Supplement: Supplementary file 1 — Supporting File: ansa70055‐sup‐0001‐SuppMat.docx. [file ANSA-6-e70055-s001.docx]

**Supporting Information**

**Integrated Extraction Optimization and HPLC-based Quality Evaluation of Thiophenes from *Tagetes erecta* Roots**

Shuo Tian^1^, Sainan Li^1^, Jisu Park^1^, Jong-Sup Bae^2^, MinKyun Na^1,*^

*^1^College of Pharmacy, Chungnam National University, 99 Daehak-ro, Daejeon 34134, Republic of Korea.*

*^2^College of Pharmacy, Kyungpook National University, Daegu 41566, Republic of Korea*

Correspondence: MinKyun Na (mkna@cnu.ac.kr)

**
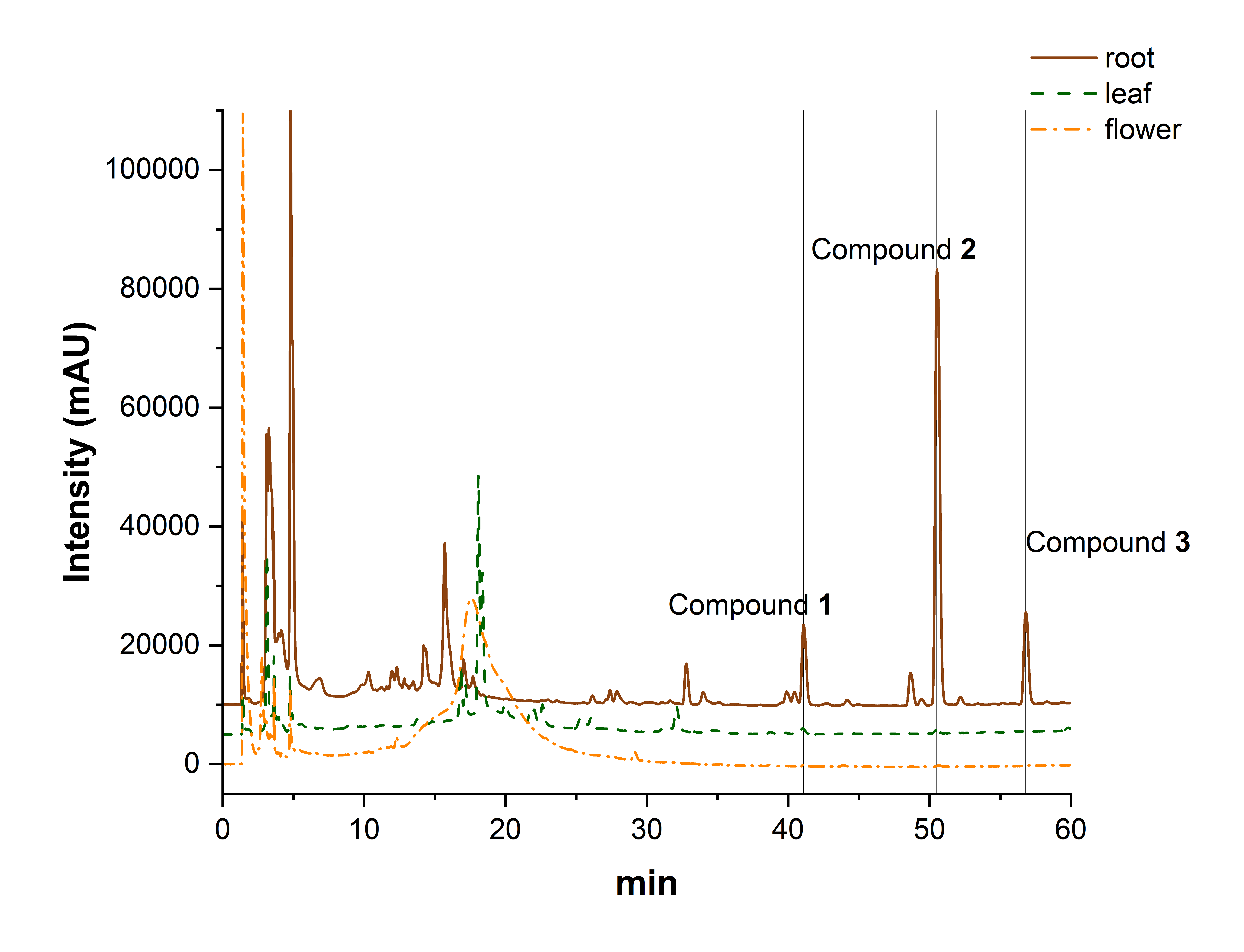
**

**Figure S1.** HPLC chromatograms of *T. erecta* flower, leaf, and root extracts obtained by reflux extraction


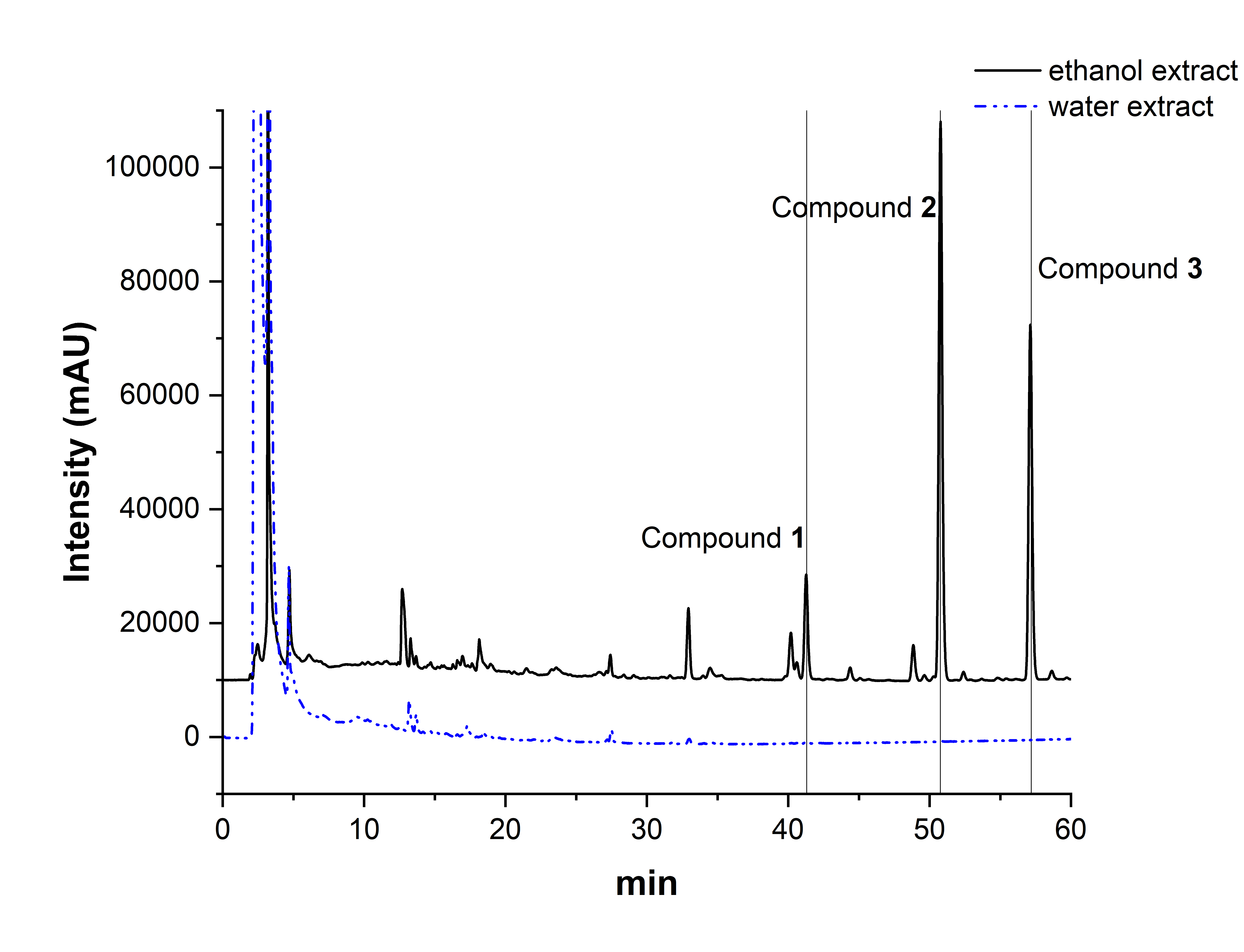


**Figure S2.** Comparative HPLC chromatograms of *T. erecta* root extracts obtained by reflux extraction using water and ethanol


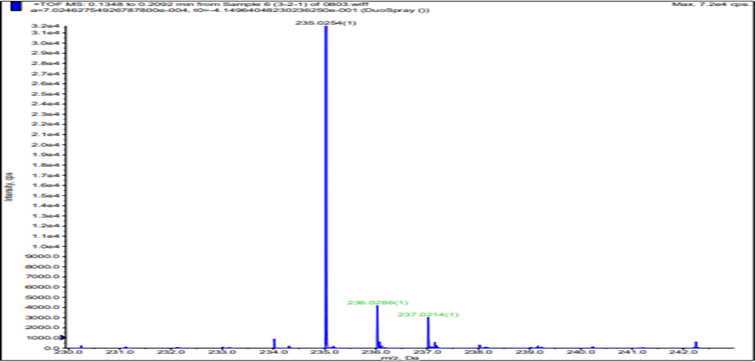


**Figure S3.** ESI-MS spectrum of compound **1**


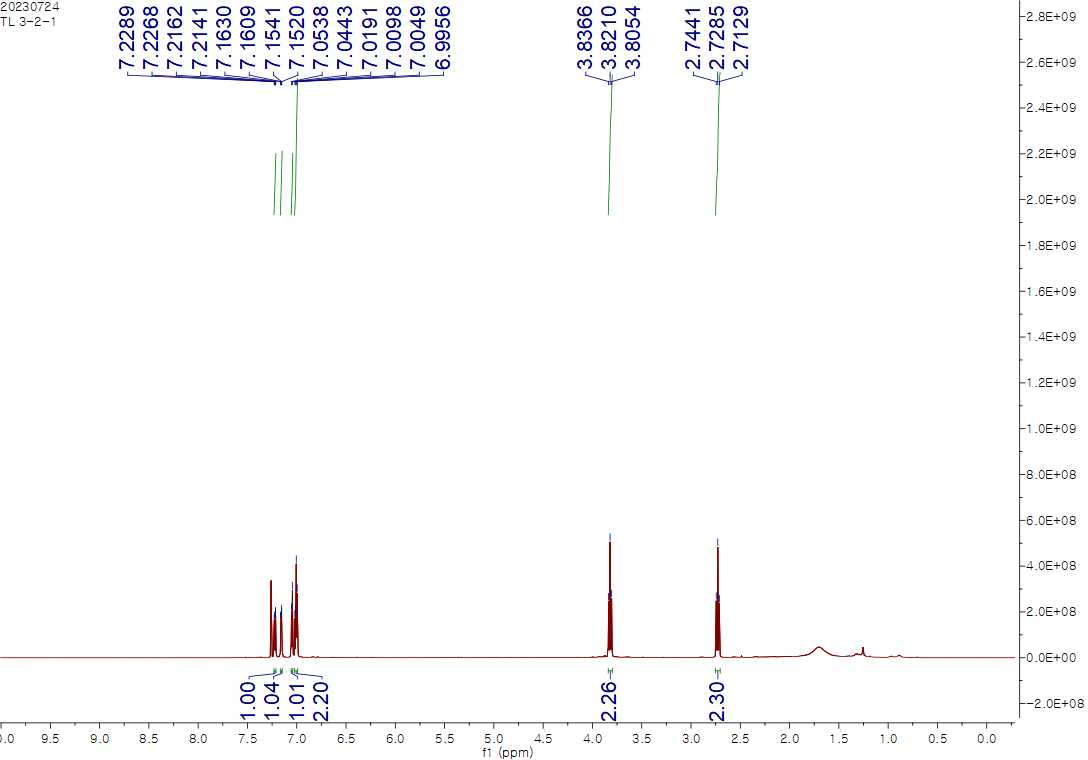


**Figure S4.** ^1^H NMR spectrum of compound **1** in CDCl_3_ (400 MHz)


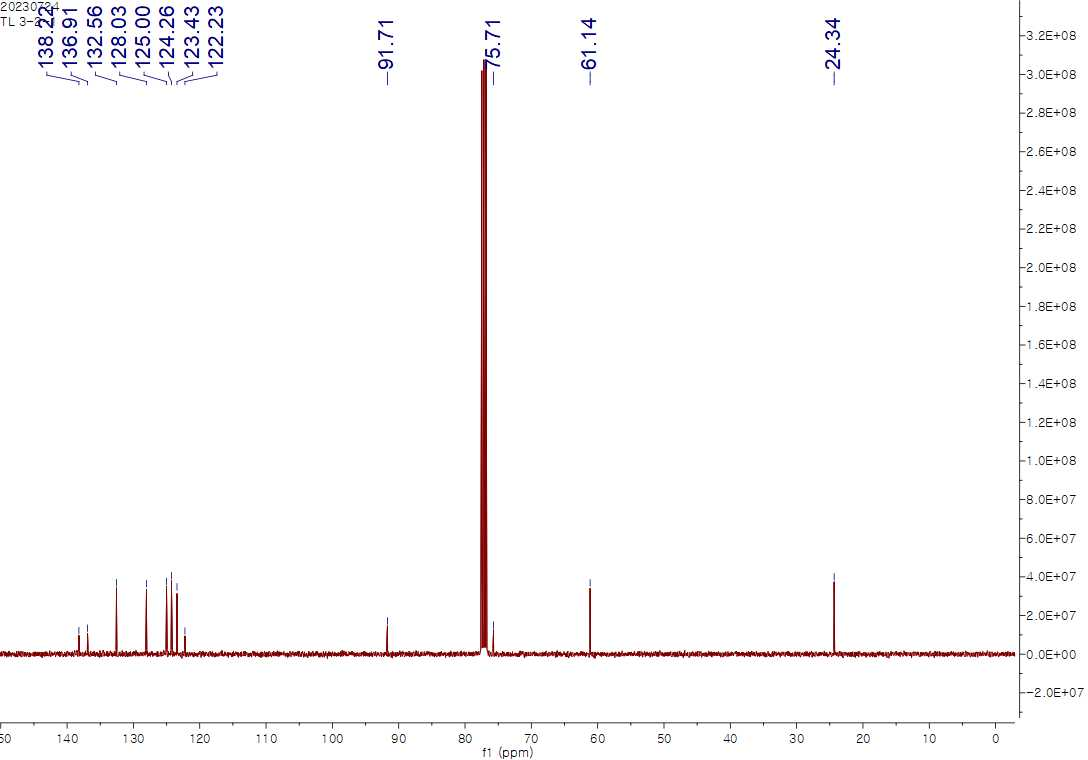


**Figure S5.** ^13^C NMR spectrum of compound **1** in CDCl_3_ (100 MHz)


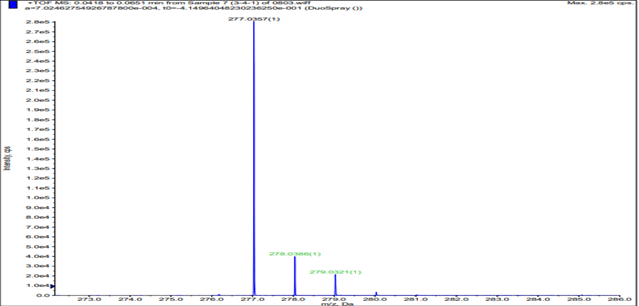


**Figure S6.** ESI-MS spectrum of compound **2**


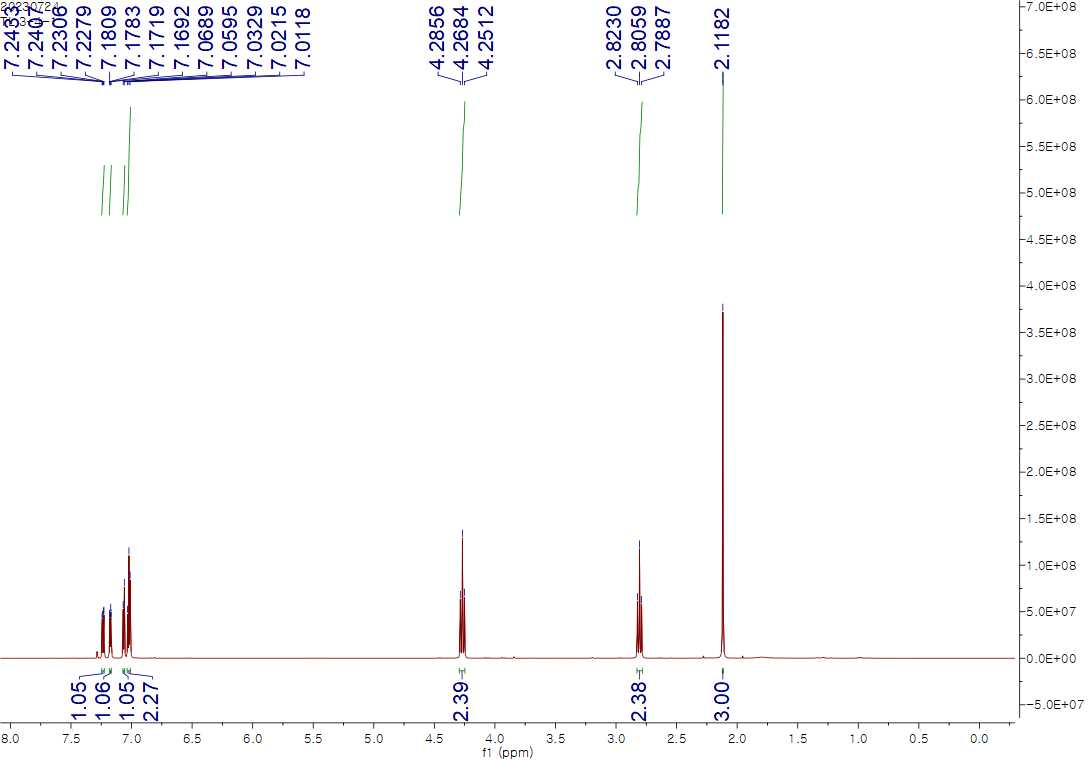


**Figure S7.** ^1^H NMR spectrum of compound **2** in CDCl_3_ (400 MHz)


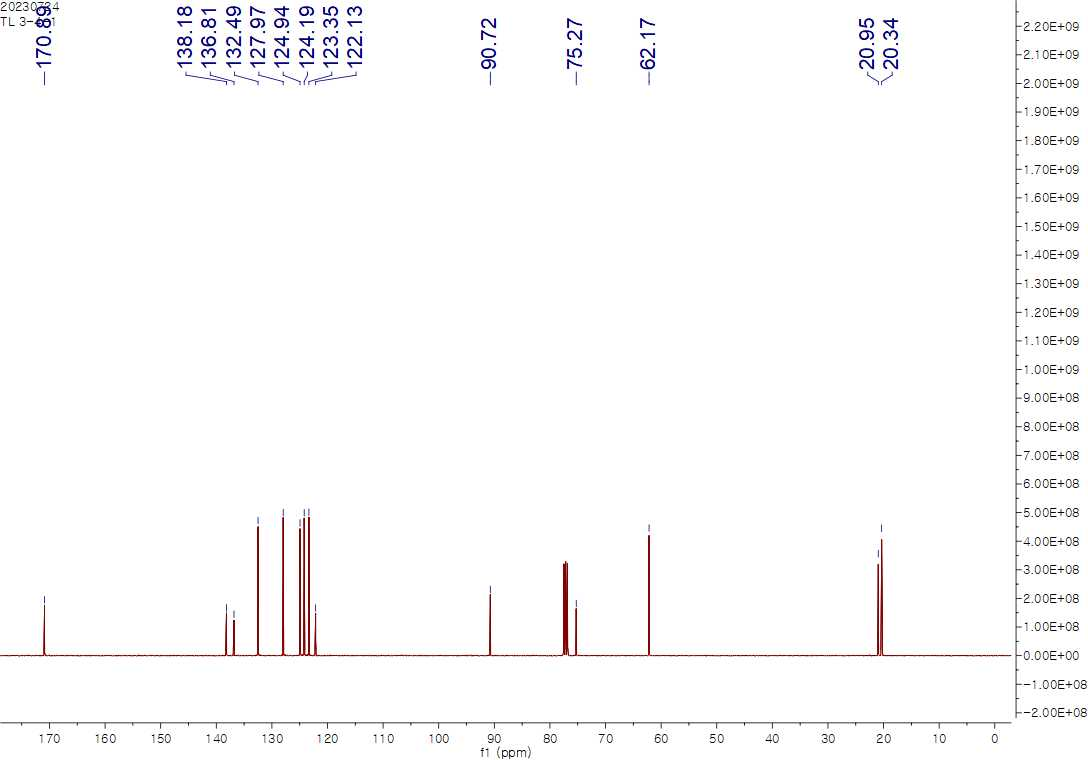


**Figure S8.** ^13^C NMR spectrum of compound **2** in CDCl_3_ (100 MHz)


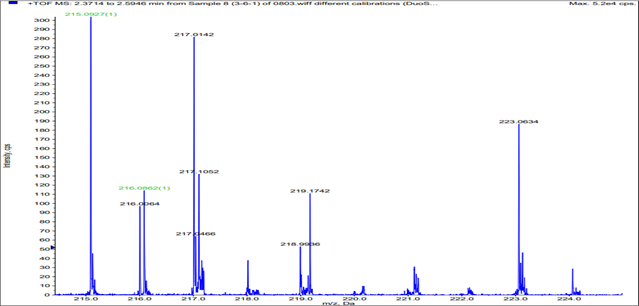


**Figure S9.** ESI-MS spectrum of compound **3**


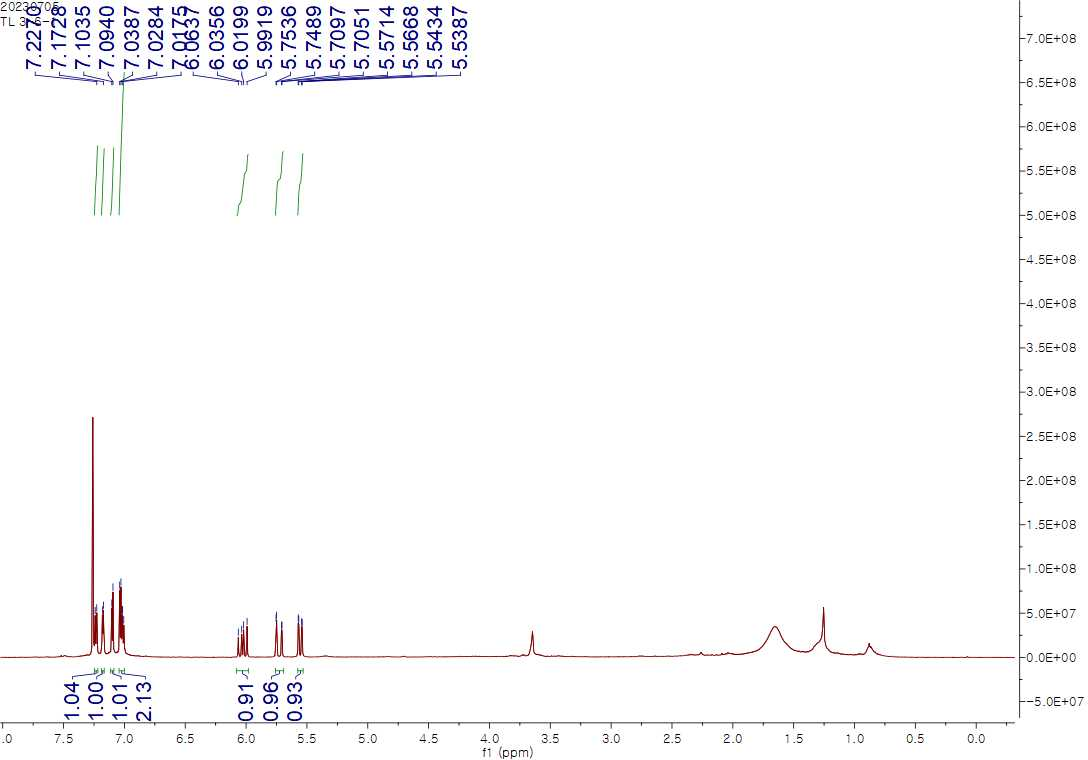


**Figure S10.** ^1^H NMR spectrum of compound **3** in CDCl_3_ (400 MHz)


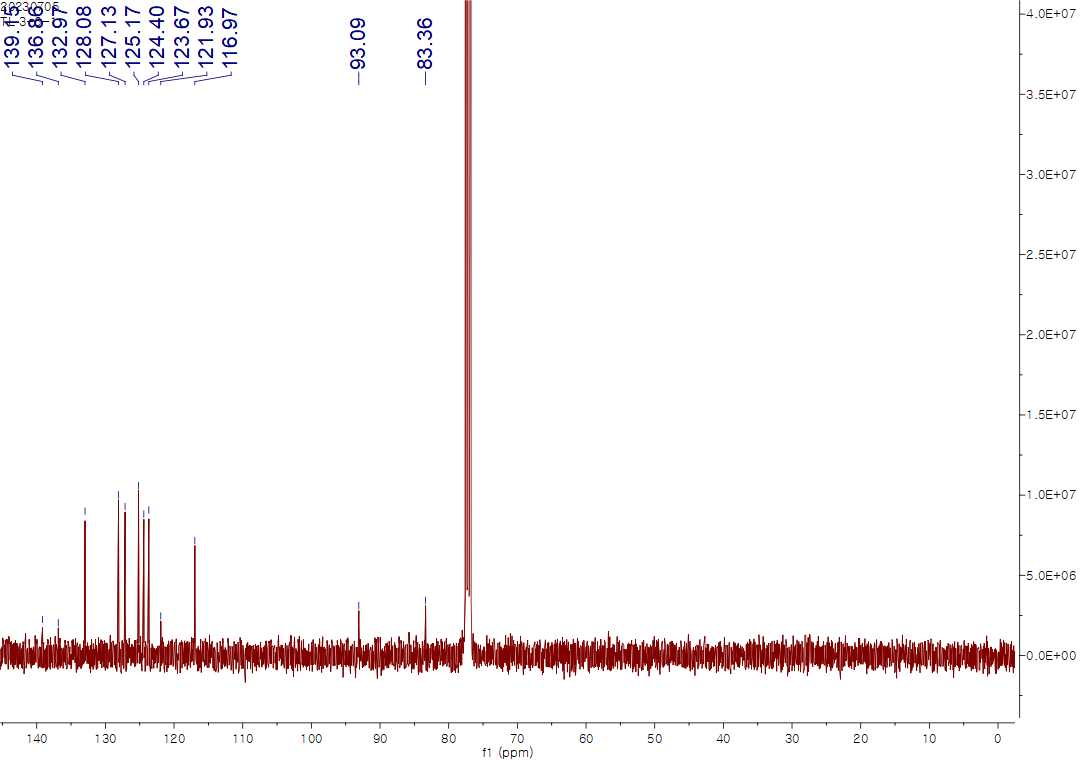


**Figure S11.** ^13^C NMR spectrum of compound **3** in CDCl_3_ (100 MHz)

**Table S1.** ^1^H (400 MHz) and ^13^C (100 MHz) NMR chemical shifts (*δ* in ppm) of compounds **1–3** in CDCl_3_

|  |  | **1** | |  | **2** | |  | **3** | |
| --- | --- | --- | --- | --- | --- | --- | --- | --- | --- |
| no. |  | *δ*_C_, type | *δ*_H_ mult. (*J* in Hz) |  | *δ*_C_, type | *δ*_H_ mult. (*J* in Hz) |  | *δ*_C_, type | *δ*_H_ mult. (*J* in Hz) |
| 2 |  | 138.2, C | - |  | 138.2, C | - |  | 139.2, C | - |
| 3 |  | 123.4, CH | 7.01, dd (3.7, 5.7) |  | 123.4, CH | 7.02, t (4.2) |  | 123.7, CH | 7.02, m |
| 4 |  | 132.6, CH | 7.01, dd (3.7, 5.7) |  | 132.5, CH | 7.02, t (4.2) |  | 133.0, CH | 7.10, d (3.8) |
| 5 |  | 122.2, C | - |  | 122.1, C | - |  | 121.9, C | - |
| 6 |  | 75.7, C | - |  | 75.3, C | - |  | 83.4, C | - |
| 7 |  | 91.7, C | - |  | 90.7, C | - |  | 93.1, C | - |
| 8 |  | 24.3, CH_2_ | 2.73, t (6.3) |  | 21.0, CH_2_ | 2.81, t (6.9) |  | 117.0, CH | 6.03, dd (17.5, 11.2) |
| 9 |  | 61.1, CH_2_ | 3.82, t (6.3) |  | 62.2, CH_2_ | 4.27, t (6.9) |  | 127.1, CH_2_ | 5.73, dd (17.5, 1.9)  5.56, dd (11.2, 1.9) |
| 11 |  |  |  |  | 170.9, C | - |  |  |  |
| 12 |  |  |  |  | 20.3, CH_3_ | 2.12, s |  |  |  |
| 2' |  | 136.9, C | - |  | 136.8, C | - |  | 136.9, C | - |
| 3' |  | 124.3, CH | 7.16, dd (3.6, 0.9) |  | 124.2, CH | 7.18, dd (3.6, 1.1) |  | 124.4, CH | 7.18, d (3.2) |
| 4' |  | 128.0, CH | 7.05, d (3.6) |  | 128.0, CH | 7.06, d (3.6) |  | 128.1, CH | 7.02, m |
| 5' |  | 125.0, CH | 7.22, dd (5.1, 0.9) |  | 124.9, CH | 7.24, dd (5.1, 1.1) |  | 125.2, CH | 7.23, d (5.1) |

**Table S2.** Stability of standard solutions of compounds **1**–**3** stored at 4 °C for 24 h and 48 h, expressed as %Remaining and RSD (n = 3)

| Compound | Concentration (µg/mL) | Condition | Mean Area (n=3) | %Remaining | RSD (%) |
| --- | --- | --- | --- | --- | --- |
| 1 | 5.0 | day 0 | 1041900.00 | 100.00 | 0.32 |
|  |  | 24 h | 1049553.00 | 100.73 | 0.17 |
|  |  | 48 h | 1048406.00 | 100.62 | 0.21 |
| 2 | 60.0 | day 0 | 3329157.00 | 100.00 | 1.28 |
|  |  | 24 h | 3211953.67 | 96.48 | 0.14 |
|  |  | 48 h | 3230602.67 | 97.04 | 0.08 |
| 3 | 40.0 | day 0 | 2758986.67 | 100.00 | 0.14 |
|  |  | 24 h | 2707872.00 | 98.15 | 0.52 |
|  |  | 48 h | 2731509.00 | 99.00 | 1.18 |

|  | Room Temp 3 d | Reflux 1 h | Reflux 2 h | Reflux 3 h | Ultrasonic 1 h | Ultrasonic 2 h | Ultrasonic 3 h | Ultrasonic 30 min | Similarity Index |
| --- | --- | --- | --- | --- | --- | --- | --- | --- | --- |
| Room Temp 3 d | 1 | 1 | 0.993 | 0.992 | 0.990 | 0.987 | 0.993 | 0.999 | 0.994 |
| Reflux 1 h | 1 | 1 | 0.995 | 0.995 | 0.994 | 0.991 | 0.996 | 0.999 | 0.997 |
| Reflux 2 h | 0.993 | 0.995 | 1 | 1 | 1 | 0.999 | 1 | 0.990 | 1 |
| Reflux 3 h | 0.992 | 0.995 | 1 | 1 | 1 | 0.999 | 1 | 0.989 | 1 |
| Ultrasonic 1 h | 0.990 | 0.994 | 1 | 1 | 1 | 1 | 1 | 0.987 | 1 |
| Ultrasonic 2 h | 0.987 | 0.991 | 0.999 | 0.999 | 1 | 1 | 0.999 | 0.984 | 0.999 |
| Ultrasonic 3 h | 0.993 | 0.996 | 1 | 1 | 1 | 0.999 | 1 | 0.991 | 1 |
| Ultrasonic 30 min | 0.999 | 0.999 | 0.990 | 0.989 | 0.987 | 0.984 | 0.991 | 1 | 0.992 |
| Similarity Index | 0.994 | 0.997 | 1 | 1 | 1 | 0.999 | 1 | 0.992 | 1 |

**Table S3.** Similarity matrix of *T. erecta* root extracts obtained under 13 extraction conditions, calculated from Pearson correlation coefficients (*r*) of 13 common characteristic peaks
